# Brain-Gut-Microbiota Axis in Amyotrophic Lateral Sclerosis: A Historical Overview and Future Directions

**DOI:** 10.14336/AD.2023.0524

**Published:** 2024-02-01

**Authors:** Shilan Chen, Xinhong Cai, Lin Lao, Yuxuan Wang, Huanxing Su, Haitao Sun

**Affiliations:** ^1^Clinical Biobank Center, Microbiome Medicine Center, Department of Laboratory Medicine, Zhujiang Hospital, Southern Medical University, Guangzhou 510280, China.; ^2^Neurosurgery Center, The National Key Clinical Specialty, The Engineering Technology Research Center of Education Ministry of China on Diagnosis and Treatment of Cerebrovascular Disease, Guangdong Provincial Key Laboratory on Brain Function Repair and Regeneration, The Neurosurgery Institute of Guangdong Province, Zhujiang Hospital, Southern Medical University, Guangzhou 510280, China.; ^3^Key Laboratory of Quality Research in Chinese Medicine, Institute of Chinese Medical Sciences, University of Macau, Taipa, Macau.; ^4^Key Laboratory of Mental Health of the Ministry of Education, Guangdong-Hong Kong-Macao Greater Bay Area Center for Brain Science and Brain-Inspired Intelligence, Southern Medical University, Guangzhou, China.

**Keywords:** Amyotrophic lateral sclerosis, brain-gut-microbiota axis, gut microbiota, potential treatment, short-chain fatty acid, microglia

## Abstract

Amyotrophic Lateral Sclerosis (ALS) is a devastating neurodegenerative disease which is strongly associated with age. The incidence of ALS increases from the age of 40 and peaks between the ages of 65 and 70. Most patients die of respiratory muscle paralysis or lung infections within three to five years of the appearance of symptoms, dealing a huge blow to patients and their families. With aging populations, improved diagnostic methods and changes in reporting criteria, the incidence of ALS is likely to show an upward trend in the coming decades. Despite extensive researches have been done, the cause and pathogenesis of ALS remains unclear. In recent decades, large quantities of studies focusing on gut microbiota have shown that gut microbiota and its metabolites seem to change the evolvement of ALS through the brain-gut-microbiota axis, and in turn, the progression of ALS will exacerbate the imbalance of gut microbiota, thereby forming a vicious cycle. This suggests that further exploration and identification of the function of gut microbiota in ALS may be crucial to break the bottleneck in the diagnosis and treatment of this disease. Hence, the current review summarizes and discusses the latest research advancement and future directions of ALS and brain-gut-microbiota axis, so as to help relevant researchers gain correlative information instantly.

## 1. Introduction

Amyotrophic lateral sclerosis (ALS) is a neurodegenerative disease characterized primarily by progressive and painless muscle weakness for the death of upper and lower motor neurons [1, 2]. The clinical manifestations of ALS can be divided into two categories: non-motor symptoms and motor symptoms. Non-motor symptoms mainly include frontotemporal dementia and cognitive impairment, while motor symptoms include dysphagia, dyspnea, and upper and lower limbs muscle weakness [1, 3].

Age and region are closely associated with the incidence of ALS. Studies show that the incidence of ALS increases gradually from the age of 40, peaks at 65 to 70, and then declines sharply [4]. Accordingly, age may play a significant role in ALS development. Furthermore, ALS was considered a relatively rare condition previously, but given factors such as the progress of aging population and the improvement of diagnostic methods, the incidence of ALS may show an increasing trend in the coming decades [3, 5]. There are also obvious regional differences in the incidence of ALS. According to statistics, the incidence in Europe was 2.2/1000000, higher than that in East Asia (0.89/10000) and South Asia (0.79/1000000) [6]. When latitude is taken as the reference, researchers found that the incidence in the northern population is higher than that in the southern [7]. The most common mutation of ALS is the C9orf72 gene, and some scholars believe that the mutation first occurred in ScandiNavia peninsula and spread outward from there [8]. Therefore, the incidence of ALS varies by region and may be the result of a combination of gene mutations and population migration. In addition, the onset of ALS may also closely relate to exercise. Several researches have demonstrated that most ALS patients have a high level of exercise intensity before the onset of the disease [9]. To be more specifically, the incidence of ALS may relate to the sports selected by the patients [10]. Scarmeas and his colleagues proposed that ALS is more prevalent among football players than among other athletes, and the risk of death is about 6.5 times that of other ALS athletes [11].

**Figure 1.**
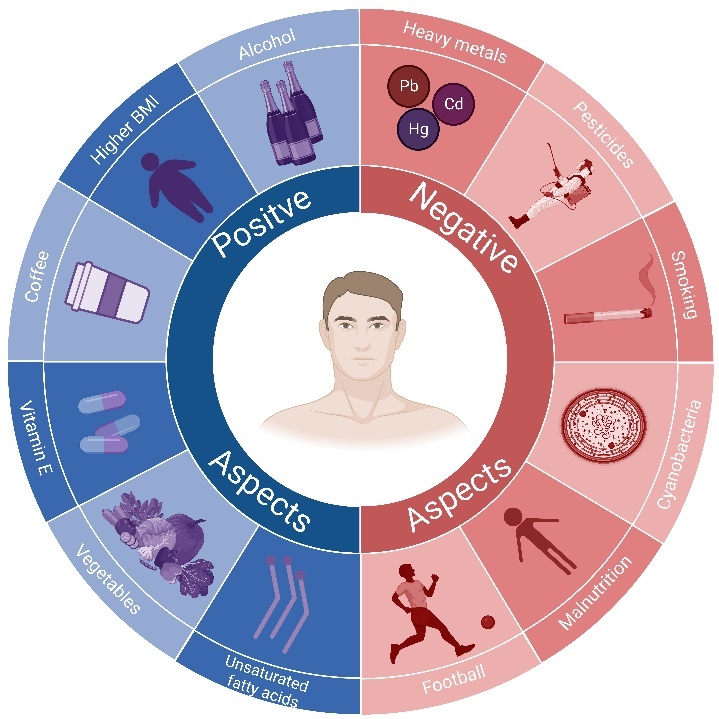
**Potential positive and negative aspects for the onset and progression of ALS**. Many factors are thought to influence the onset and progression of ALS. Among them, positive aspects such as higher alcohol consumption, higher BMI and active intake of Vitamin E are believed to delay the progression of the disease and are associated with longer survival. In contrast, negative aspects such as exposure to heavy metals and pesticides, smoking and playing football are thought to accelerate the progression of the disease and are closely related to poor prognosis.

According to statistics, most patients die within 3-5 years of onset [12, 13]. However, there are also patients who live longer. A review showed that up to 10% of ALS patients survived for more than eight years after the onset [14]. Turner et al. found that the survival rate for patients over ten years was 4% [12]. There are many factors that can affect the survival time of patients, such as the location of lesion, gender, age, diet and environment [6, 15, 16]. Interestingly, the progression of ALS may also be strongly influenced by nutritional factors. Patients with ALS generally suffer from malnutrition, which is mainly caused by insufficient nutrition intake and exuberant metabolism [17, 18]. Malnutrition negatively affects neuromuscular status, which in turn is exacerbated by insufficient nutrition intake due to poor muscle status in ALS patients. In addition, we also summarized other potential positive and negative factors that may affect the occurrence and progression of ALS (Fig. 1) [9, 19-23]. It should be noted that there is still a lack of consensus on the role of certain factors in ALS. Therefore, the potential positive and negative factors of ALS are also one of the topics that need to be further explored in the future.

**Figure 2.**
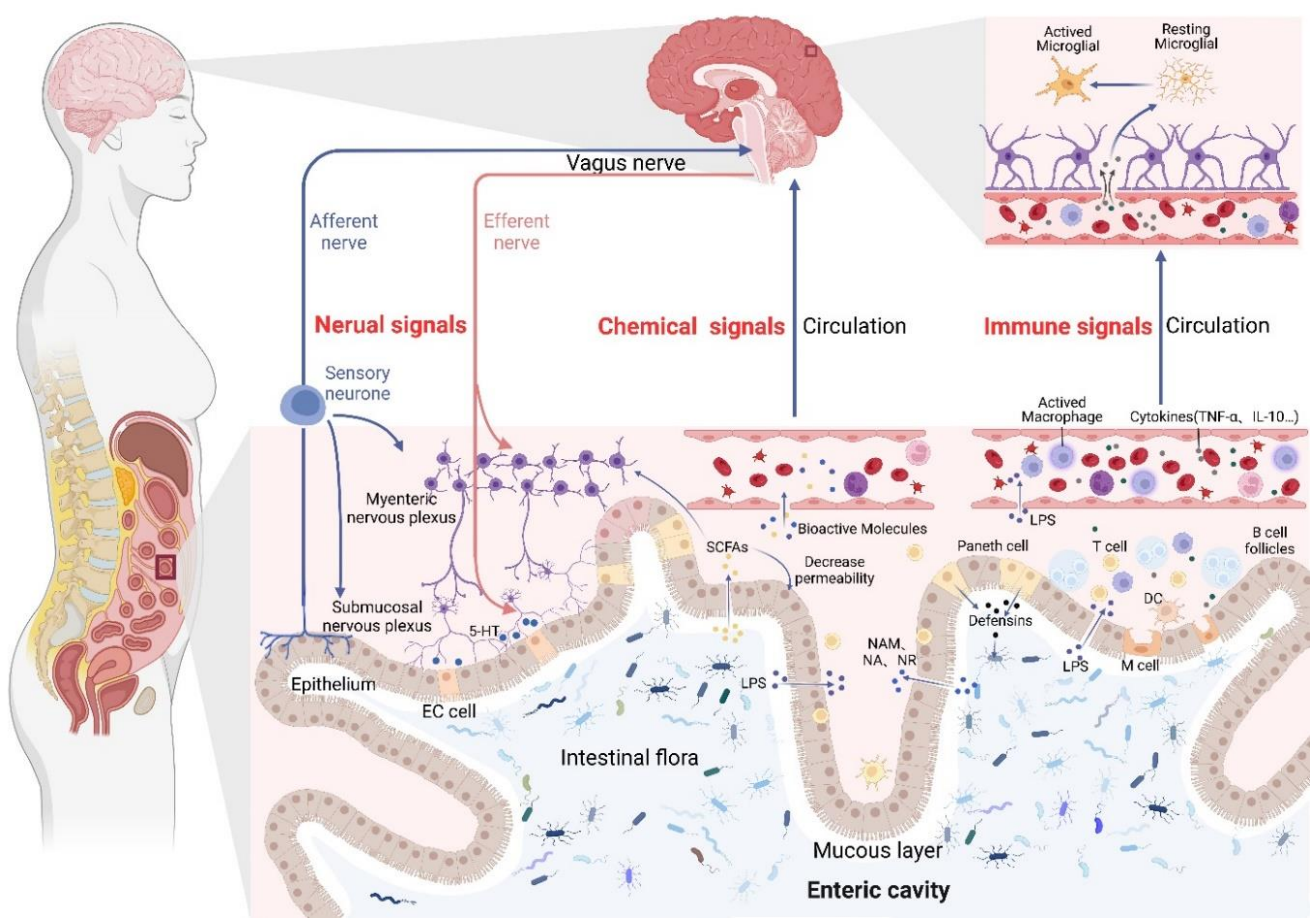
**Potential role of the brain-gut-microbiota axis in ALS**. There is a two-way interaction between the brain-gut-microbiota axis. It acts in many ways, including immune, nervous, and endocrine mechanisms. In the immune mechanism, the gut microbiota can affect the CNS through two links of peripheral inflammation and central inflammation, in which LPS plays an important role. LPS can activate monocytes and macrophages in peripheral blood to induce the release of a large number of pro-inflammatory cytokines. In addition, TNF-α and IL-10 also play an important role in the immune mechanism. In the neural mechanism, gut microbiota signals can communicate bidirectionally with the CNS through ANS and ENS, and the mainly functional parts include vagus nerve, Paneth cell, and ENS. As for endocrine mechanism, small molecular substances from various bacteria, including SCFAs, NAM, NA, NR and others, play an important role in the development of ALS disease. They can enter the circulatory system through the blood and affect CNS. Any abnormality in any part of any one mechanism may cause the disorder of the whole brain-gut-microbiota axial circuit.

The dysbiosis of gut microbiota and the disorder of brain-gut-microbiota axis are thought to play a significant role in many neurodegenerative diseases [24-26]. *Firmicutes* and *Bacteroides* make up the majority of the gut microbiota, which help in maintaining intestinal function [27]. Gut microbiota is also involved in absorbing and storing nutrients, converting metabolites, maintaining intestinal barrier integrity and participating in immune responses [28]. The concept of brain-gut-microbiota axis was put forward and gradually improved in the 1840s. It mainly describes the information interaction process between the gut microbiota and the brain from three aspects: nerves, immunity and endocrine [29, 30]. Despite extensive researches have been done, the cause and pathogenesis of ALS remains unclear. However, many research results in recent years show that dysbiosis of gut microbiota may contribute to the occurrence and development of ALS. Through the brain-gut-microbiota axis, signals from the brain of patients with ALS can shape the gut microbiota (Fig. 2). Similarly, brain function can also be affected by gut microbial information [24, 31]. Therefore, improving the dysbiosis of gut microbiota may delay the progression of ALS and prolong the survival time of patients.

According to the research results of the brain-gut-microbiota axis, scientists have proposed many therapeutic strategies for gut microbiota, such as fecal microbial transplantation (FMT), supplementing probiotics, prebiotics, synbiotics and postbiotics. Current review aims to summarize the latest research results related to ALS, gut microbiota and brain-gut-microbiota axis, clarifying the mechanism of action between gut microbiota and brain. We hope that this review can help scholars in related fields to quickly grasp the research progress related to the disease, find new research ideas and promote the development of ALS treatment.

## 2. Dysbiosis of gut microbiota in ALS

With the problem of population aging becoming more and more prominent, research on aging is also deepening. In the latest research, the dysbiosis of gut microbiota is considered one of the twelve characteristics of aging, revealing the close correlation between aging and gut microbiota [32]. The composition and diversity of gut microbiota such as the ratio of *Firmicutes/Bacteroides* will change with age increased [33, 34]. Dysbiosis of gut microbiota was also observed in two mouse models of progeria [35].At the meantime, dysbiosis of gut microbiota will also affect aging process. After transplanting the fecal microbiota of the aged mice into the young mice, the quantity of pro-inflammatory cytokines and activated microglia in the young mice were increased, and aging conditions such as age-related neuroinflammation also occurred in the young mice[36]. On the contrary, after the fecal microbiota of young mice were transplanted into old mice, the aging condition of old mice was significantly improved [36]. ALS, as an age-related neurodegenerative disease, is also closely related to the gut microbiota. In a study of SOD1^G93A^ mice, it was found that the progression of ALS can change the gut microbiota, thus influencing the life span of mice [37]. Therefore, studying the gut microbiota of patients with ALS may help us to better understand ALS.

**Table 1:** Dysbiosis of gut microbiota in ALS.

	Experimental subject		Conclusion	Ref.
	Animal model	Human volunteer		
Ning et al	_	20,806 cases with ALS and 59,804 controls (GWAS summary statistics from IALSC); 18,340 participants (GWAS summary statistics from the MiBioGen); 7824 participants (GWAS summary statistics from TwinsUK and KORA)	Decreased abundance of *Fusicatenibacter* and *Catenibacterium*; increased abundance of *Lachnospira*; these changes may be related to the higher risk of ALS	[44]
Zhang et al	_	20,806 cases with ALS and 59,804 controls (GWAS summary statistics from IALSC); 1812 sanples (GWAS summary statistics); 7824 adult individuals (GWAS summary statistics from 2 European cohorts)	Increased abundance of *OTU4607 Soutella* and *Lactobacillales order*; this change may be correlated with the genetically predicted increased susceptibility to ALS	[45]
Zeng et al	_	8 females and 12 females diagnosed with ALS; 8 females and 12 females matched controls	Up-regulation of *Bacteroidetes*, *Kineothrix*, *Parabacteroides*, *Odoribacter*, *Sporobacter*, and *unclassified Porphyromonadaceae*	[40]
Hertzberg et al	_	10 patients diagnosed with ALS (3 females and 7 males) and their opposite sex spouses (7 females and 3 males); 10 opposite sex couples' healthy controls	Compared with their spouses, down-regulation of *Prevotella spp* and *P.timonensis*; up-regulation of species richness and uniformity of gut microbiota.	[46]
Figueroa-Romero et al	SOD1(G93A) mice	_	Down-regulation of *Akkermansia*, *Coriobacteriaceae* and *Adlercreutzia*	[37]
Nicholson et al	_	26 females and 40 males diagnosed with ALS; 36 females and 25 males' healthy controls; 7 females and 5 males' Neurodegenerative controls	Decreased abundance of *Roseburia intestinalis* and *Eubacterium rectale* (two major butyrate-producing bacteria); decreased abundance of eight major butyrate-producing bacteria	[47]
Fang et al	_	6 patients diagnosed with ALS; 5 healthy controls	Decreased abundance of *Anaerostipes*, *Lachnospiraceae* (two butyrate-producing bacteria) and *Oscillabacter*; Increased abundance of *Dorea*	[41]
Zhai et al	_	4 females and 4 males diagnosed with ALS; 4 females and 4 males' healthy controls	Decreased abundance of *Faecalibacterium* (one butyrate-producing bacteria)	[43]
Zhang et al	SOD1(G93A) mice	_	Decreased abundance of *Lachnospiraceae* (one butyrate-producing bacteria) occurred at the early stage of ALS disease	[48]
Wu et al	SOD1(G93A) mice	_	Decreased abundance of *Butyrivibrio Fibrisolvens* (one butyrate-producing bacteria)	[49]

More and more studies show that ALS is related to the dysbiosis of gut microbiota, and there are many differences between the ALS patients and healthy individuals in gut microbiota (Table 1). In the gastrointestinal tract, *Firmicutes* mainly produces butyrate, while *Bacteroides* mainly produces propionate [38], the ratio between them is closely related to human health [39]. The ratio of *Firmicutes* to *Bacteroidetes* commonly show a decline in ALS patients [40-42]. However, some studies have also pointed out that the ratio of *Firmicutes* to *Bacteroides* in ALS patients shows an increasing trend [43]. Studies have also shown that the decrease of *Fusicatenibacter* and *Catenibacterium* and the increase of *Lachnospira* in patients may associate with the higher risk of ALS [44]. Moreover, the increase in the relative abundance of *OTU4607 Soutella* and *Lactobacillales order* in ALS patients was also correlated with the genetically predicted increased susceptibility to ALS [45]. The bacteria such as *Bacteroidetes*, *Odoribacter*, *Kineothrix*, *Sporobacter, Parabacteroides* and *unclassified Porphyromonadaceae* are significantly increased in the intestinal tract of ALS patients [40]. Interestingly, a research found that the richness and uniformity of gut microbiota in ALS patients were obviously higher than their spouses, except for *Prevotella spp* and *P.timonensis* [46]. On the animal model, an experiment on SOD1^G93A^ mice found that the quantity of *Akkermansia*, *Coriobacteriaceae* and *Adlercreutzia* in the intestine was low, indicating that the gut microbiota of ALS animals will change as well [37].

Of note, butyrate-producing bacteria showed reduced abundance in both ALS patients and animal models. In ALS patients, the abundance of *Roseburia intestinalis* and *Eubacterium rectale* was significantly reduced, which are two major butyrate-producing bacteria. Besides, the total relative abundance of eight main butyrate-producing bacteria was also obviously reduced [47]. Moreover, a high-throughput sequencing study showed that the relative abundance levels of two butyrate-producing bacteria, *Anaerostipes* and *Lachnospiraceae*, and genus *Oscillabacter* in the intestinal tract of ALS patients were significantly lower than those in the healthy control group, while the relative abundance of the harmful bacterium, *Dorea*, was significantly increased [41]. Markedly decreased of *Faecalibacterium* (a butyrate-producing bacterium) was also observed in patients with ALS [43]. The decrease of butyrate-producing bacteria was also observed in ALS animal model research. The butyrate-producing bacteria such as *Lachnospiraceae* in the intestinal tract of SOD1^G93A^ mice were also significantly reduced, and the change of gut microbiota occurred at the early stage of ALS, even earlier than the deterioration of locomotor ability [48]. Similarly, the decrease of *Butyrivibrio Fibrisolvens* was also found in another group of SOD1^G93A^ mice in the early phase of symptoms [49].

The experimental results in many animal models of ALS indicate that the dysbiosis of gut microbiota will further worsen ALS diseases. Compared with the SOD1^G93A^ mice not treated with antibiotics, the SOD1^G93A^ mice treated with antibiotics showed poorer locomotor ability and more severe ALS disease progression [50]. Not only that, researchers inoculated 11 microorganisms which were altered in ALS patients into SOD1^G93A^ mice, and claimed that the disease progression in SOD1^G93A^ mice was aggravated after *P. dasonis* and *R.torques* were transplanted [50]. Meanwhile, the dysbiosis of gut microbiota is closely concerned with the increase of intestinal microbial translocation. Plasma lipopoly-saccharide-binding protein (LBP) levels can reveal whether microbiota have entered the blood. And the obvious increase of LBP level in sALS patients indicates that dysbiosis of gut microbiota might promote intestinal microbial translocation in patients with sALS and thus affect the progression of sALS disease [51]. It is worth mentioning that at present, most experimental studies on antibiotic treatment of ALS animal models are conducted using SOD1^G93A^ mice. And treatment of SOD1^G93A^ mice with antibiotics often worsened the disease. On the contrary, antibiotic treatment for C9orf72 mutant mice can delay the progression of ALS disease [52].

**Table 2:** Barrier Dysfunction in ALS.

	Experimental subject		Conclusion	Ref.
	Animal model	Human volunteer		
Intestinal barrier			
Wu et al	SOD1(G93A) mice	_	Decreased expression of ZO-1,E-Cadherin-1 and lysozyme 1; increased expression of haptoglobin and abnormal Paneth cells	[49]
Zhang et al	_	7 females and 16 males diagnosed with sALS (mean age 59.2 ± 8.7 years); 6 females and 12 males' healthy donors (mean age 54.5 ± 8.4 years); 11 females and 7 males diagnosed with AD (mean age 78.9 ± 8.3 years)	High expression of plasma LPS, increasing with the aggravation of clinical symptoms	[53]
Blood-spinal cord barrier			
Garbuzova-Davis et al	SOD1(G93A) mice	_	Intracellular and extracellular edema of blood vessels; high degree of vacuolation and degenerative damage of endothelial cells	[55]
Nicaise et al	SOD1(G93A) mice	_	Swelling and detachment of astrocyte terminal podoid from the endothelium; reduction of basement membrane component; capillary leakage; downregulation of tight junction proteins	[56]
Garbuzova-Davis et al	SOD1(G93A) mice	_	Microbleeds	[57]
Nicaise et al	SOD1(G93A) mice	_	Increased expression of AQP-4	[58]
Zhong et al	SOD1(G93A) mice SOD1(G37R) mice SOD1(G85R) mice	_	Decreased expression of ZO-1, Occudin, Claudin-5, Glut-1, total length of capillaries, blood flow to the spinal cord; microbleeds and hemosiderin deposits in the spinal cord parenchyma	[60]
Garbuzova-Davis et al	_	12 males and 13 females diagnosed with ALS (mean age 64.8 ± 1.90 years); 13 males and 5 females’ controls (59.3 ± 3.78 years)	Degeneration of pericytes around the blood-spinal cord barrier; dilation of type IV collagen in the perivascular basement membrane; ultrastructural abnormalities of white matter capillaries	[61]
Blood-brain barrier			
Garbuzova-Davis et al	SOD1(G93A) mice	_	High degree of vacuolation and degeneration of endothelial cells	[55]
Nicaise et al	SOD1(G93A) mice	_	Astrocytic degeneration; intracellular and extracellular edema of vascular endothelial cells; decreased levels of tight junction proteins; capillary leakage	[56]
Garbuzova-Davis et al	_	13 ALS patients (11 patients diagnosed with sALS, 2 patients diagnosed with fALS); 6 healthy controls	Decreased level of circulating endothelial cells in the peripheral blood	[67]
Boston-Howes et al	SOD1(G93A) mice SOD1(B6SJL) mice	_	Increased expression of P-glycoprotein in spinal cord	[73]
Chan et al	SOD1(G93A) mice	_	With the progression of ALS, the transporter activity of P-glycoprotein is increasing, and the expression level is also increasing	[72]
Garbuzova-Davis et al	_	_	The composition of the basement membrane is changed; the basement membrane metalloproteases MMP-2 and MMP-9 are activated	[68]

## 3. Barrier dysfunction in ALS

There are many important "barriers" in the human body to maintain the microenvironment balance in different regions of the body, which are crucial for the health of the body. At present, many animal experiments and patient data of ALS have shown that the intestinal barrier, blood-brain barrier and blood-spinal cord barrier have different degrees of dysfunction, and the destruction of barrier function occurs before motor neuron degeneration and inflammatory reaction (Table 2).

### 3.1 Intestinal barrier

The intestinal barrier consists of mucus layer, epithelial barrier and intestinal vascular barrier. It can effectively regulate and control the nutrient substance in the intestinal tract to enter the blood, and simultaneously prevent the harmful substance from entering. It also plays a good barrier function by participating in the regulation of gut microbiota and intestinal secretion.

Currently, many experimental results have reported that the integrity of intestinal barrier is broken in ALS animal models and patients. For instance, decreased expression of ZO-1, E-Cadherin and increased haptoglobin were observed in the SOD1^G93A^ mice, showing impaired tight junction and adhesive junction [49]. Besides, abnormal Paneth cells increased and lysozyme 1 decreased in SOD1^G93A^ mice, which may induce autophagy dysfunction [49]. The decreased function of the body to eliminate misfolded proteins will further lead to intestinal dysfunction. The same study also proved that the intestinal uptake of FITC dextran was increased in ALS model mice, which means the increased intestinal permeability. At the same time, it was also proved that patients with sALS have a high level of Lipopolysaccharide (LPS) [53]. Physiological concentration of LPS can lead to impaired intestinal tight junction and increased intestinal permeability through a TLR4-dependent process[54]. In addition to increasing intestinal permeability, high level of LPS can also induce intestinal inflammation through the signal transduction axes of TLR4, FAK and MyD88, further destroying the intestinal barrier [54].

### 3.2 Blood-spinal cord barrier

The blood-spinal cord barrier is a unique barrier existing between the blood and spinal cord which is made up of capillary endothelial cells, endothelial basal cells, pericytes and astrocytes. It can effectively maintain the homeostasis around the spinal cord and ensure the normal physiological environment of spinal neurons.

At present, obvious damage to the integrity of the blood-spinal cord barrier can be observed in animals and patients with ALS. Ultrastructurally, scientists observed extracellular edema, endothelial cell edema and high vacuolation of capillaries [55]. In addition, pathological changes such as astrocyte foot process swelling, reduced basement membrane components, downregulation of tight junction protein and microbleeding have also been reported [56, 57]. Extensive vascular endothelial cell edema may be associated with elevated AQP-4 [58]. Moreover, the change of AQP-4 may weaken the capacity of astrocytes to preserve water and K^+^ homeostasis in the central nervous system (CNS), resulting in swelling of astrocyte foot process [59]. Before the onset of ALS in animal models, scientists have observed a 10-15% reduction in total capillary length and a 30-45% reduction in spinal cord blood flow [60]. In addition, microbleeds and hemosiderin deposits as well as decreased levels of ZO-1, Occudin, Claudin-5 and Glut-1 in the spinal cord parenchyma were also observed [60], all of which indicated that the damage of the blood-spinal cord barrier occurred before the onset of the disease.

It is worth mentioning that peripheral cell degeneration, type Ⅳ collagen dilatation of basement membrane and abnormal ultrastructure of white matter capillaries of spinal cord also occurs in patients with ALS [61]. But none of these changes were observed in animal models. After the integrity of the blood-spinal cord barrier is damaged and spinal cord injury occurs, reactive astrocytes express type IV collagen and promote the formation of glial scars [62]. In vitro experiments of the same study, IL-1β and TGFβ-1 cytokines were proved having the ability to induce the expression of type IV collagen in astrocytes [62]. The proliferation of reactive astrocytes and the secretion of various cytokines such as TNF-α and TGFβ-1 are also important factors for inflammation and neuronal degeneration in ALS[63, 64]. After reactive astrocyte activating factors such as TNF-α, IL-1α and C1q were knocked out, the survival time of SOD1^G93A^ mice was significantly prolonged [65].

### 3.3 Blood-brain barrier

The blood-brain barrier exists not only between brain cells and plasma, but also between cerebrospinal fluid and plasma. It consists of brain microvascular endothelial cells, endothelial basement membrane, pericytes, and astrocyte perivascular foot. Similarly, it can effectively prevent macromolecular substances, harmful molecules and cells from entering the brain, regulate the beneficial substances into the brain, so as to maintain the steady state of the internal environment in the brain. At present, the integrity of the blood-brain barrier can be damaged in some animals and patients with ALS. At the ultrastructural level, the destruction of the blood-brain barrier was similar to that of the blood-spinal cord barrier. High degree of vacuolation and degeneration of endothelial cells occurs in SOD1^G93A^ mice [55]. The increase of circulating endothelial cells is a signal of endothelial injury [66]. However, the quantity of circulating endothelial cells may show a downward trend in ALS patients as a whole because the increased endothelial cells in circulation attach to the damaged cells and form multi-layered endothelial structures [67]. In addition, the structure of the basement membrane was also disrupted. Studies showed that the basement membrane metalloproteases MMP-2 and MMP-9 which are able to degrade laminin, fibronectin, and proteoglycan were activated, thus leading to basement membrane damage [68]. SOD1^G93A^ mice will survive longer without the MMP-9 [69]. Furthermore, astrocytic degeneration, intracellular and extracellular edema of vascular endothelial cells, decreased levels of tight junction proteins and capillary leakage also indicates that the integrity of the blood-brain barrier is damaged [56, 68].

Due to increased permeability, immune cells can cross the blood-brain barrier and gather in the CNS, inducing neuroinflammation or promoting neuronal apoptosis [70]. Moreover, microglia can secrete a variety of cytokines when activated by oxidative stress or inflammatory cytokines from the blood. Some cytokines are protective for nerve cells, while others are harmful. At the same time, blood-derived harmful substances can also reside in the CNS and cause toxic effects [70]. The lack of gut microbiota is related to the increase of the blood-brain barrier permeability. A study showed that the blood-brain barrier permeability was significantly higher in the sterile mice. Since then, scientists transplanted SCFAs-producing bacteria into the sterile mice and eventually found that the permeability was decreased [71].

P-glycoprotein, as an ABC transporter, is expressed in the microcapillary endothelial cells and has a wide range of substrate specificity. It is able to transport substrates from the blood-brain barrier and the blood-spinal cord barrier back to the blood, so it is the main obstacle for drug delivery to the brain and spinal cord [72]. As a drug used for treating ALS, the drug concentration of Riluzole in CNS is closely affected by P-glycoprotein. P-glycoprotein which expresses in spinal cord was increased in the SOD1 transgenic mice model [73]. Notably, recent studies found that with the deterioration of ALS animal, the expression level of P-glycoprotein in the blood-spinal cord barrier and blood-brain barrier increase, as well as its transport activity [72]. However, the absorption of riluzole in brain was significantly increased after the SOD1^G93A^ mice were injected the P-glycoprotein inhibitor Elacridar [74]. It was also found that Riluzole uptake in the brain of mice was also obviously increased when Riluzole was used in combination with P-glycoprotein inhibitor Minocycline [75]. Therefore, multiple generations of P-glycoprotein inhibitors have been developed for P-glycoprotein [76].

## 4. Brain-gut-microbiota axis

### 4.1 Neural pathway of brain-gut-microbiota axis

Changes in gut microbiota can affect CNS and enteric nervous system (ENS) [77]. Neurodevelopment is consistently affected by gut microbiota [78]. In 1-month-old SOD1^G93A^ mice, there was already an observable gut microbial disturbance in the absence of neuromuscular symptoms [48]. Although this phenomenon has not been demonstrated in human ALS patients, the results observed in animal models provide us with a new possibility that the gut microbiota may silently change and affect the neurodevelopment before the onset of ALS in susceptible individuals. Intestinal microbial signals can communicate bidirectionally with the CNS through the autonomic nervous system (ANS) and ENS [30]. ANS consists of sympathetic nervous system and parasympathetic nervous system. ENS is a neural network that exists at the interface between host and gut microbiota which consists of myenteric plexus and submucosal plexus. Although the ENS can operate completely independently of neural inputs from the spinal cord and brain, in actual physiological processes, ENS, sympathetic nervous system and parasympathetic nervous systems often cooperate to complete the information interaction between the CNS and the gut [79].

Enterochromaffin cells and vagus nerve may play an essential role in the process of transmitting intestinal microbial signals to the CNS in ALS patients. Enterochromaffin cells are bidirectional information converters between gut microbiota and enteric nerves. The intestinal lumen side of enterochromaffin cells can detect the stimulation from gut microbiota and food ingested by the host. Subsequently, the vagal afferent nerve endings located in the lamina propria can be activated by substances such as 5-HT and histamine secreted by chromaffin cells [80-82]. The vagus nerve consists of 80% afferent fibers and 20% efferent fibers, which is an important part of the ANS [30]. In a mouse experiment, researchers found that local infection of *Campylobacter jejuni* in the intestine can activate the sensory neurons of the vagus nerve, thus activating nucleus tractus solitarius (nTS) and areas related to the main visceral sensory pathways in the brain [83]. Ronchi et al. observed reduced activation of hippocampal microglia on the 15th day after injury to vagal afferents in mice [84]. This suggests that the vagus nerve may start providing warning signals to the brains of ALS susceptible persons as soon as small changes occur in the gut microbiota. And these warning signals may have long-term effects on the brain by activating chronic neuroinflammation.

Constipation caused by decreased intestinal motility is one of the common clinical manifestations of ALS [85]. Intestinal motility is mainly regulated by ANS and ENS [79]. Intrinsic sensory neurons such as Dogiel type I and type II neurons in the gastrointestinal tract provide a wide range of action sites for metabolites [79]. Metabolites such as SCFAs, chemotactic peptides and tryptamine derived from microbiota are believed to act on the ENS and then affect the intestinal transport rate [81, 86]. Therefore, we suppose that constipation symptoms in ALS patients may relate to the altered gut microbiota metabolites which can act on the ENS. In addition, ENS is very similar to the CNS in terms of structure and neurochemistry [87], but Kulkarni and his colleagues found substantial neuronal transformations and neurogenesis in adult intestinal neurons [88]. The gut microbiota of the human body is constantly changing dynamically. If the above conclusion is correct, the gut microbiota may also directly affect ENS neurons in the development stage, thereby changing the intestinal function of patients with ALS.

Not only is the function of CNS affected by the gut microbiota, but the gut microbiota is also affected by the neuromuscular dysfunction caused by ALS itself. ALS is a progressive degenerative disease of motor neurons. The chewing, swallowing and intestinal motor function of ALS patients will deteriorate to varying degrees over the course of progression. Therefore, with the progression of ALS, the gut microbiota not only needs to face external environmental pressure, but also is affected by the disease symptoms themselves. There are many factors affecting the gut microbiota. First of all, the slow food intake rate of patients with ALS and even the changed food intake mode (gastrostomy can be selected for patients with ALS due to dysphagia) may affect the type, quantity and quality of food intake [89], affecting the composition of gut microbiota. Secondly, reduced intestinal transport capacity will lead to the excessive reproduction of some gut microbiota [90]. In the condition of limited living resources, the original balance of the gut microbiota will be broken. In addition, the intestinal mucus layer is one of the main living areas of gut microbiota, and the dysfunction in secreting mucus, bicarbonate and water will also change the living environment of intestinal microorganisms and affect the composition of the microbiota. Furthermore, Macfarlane and his colleagues also proposed the possibility that ANS could directly regulate the response of gut immune cells to the gut microbiota [91].

### 4.2 Immune pathway of brain-gut-microbiota axis

The progression of ALS and the survival time of ALS patients may greatly be influenced by immune inflammatory response induced by gut microbiota. A study on C9orf72 mice showed that broad-spectrum antibiotics could effectively interfere with the inflammation and autoimmune phenotype in ALS mice before and after the onset of disease. Subsequent studies further confirmed that the therapeutic effect of antibiotics was actually achieved by changing the gut microbiota [92].

The biological signals emitted by the intestinal microbiota can finally affect the central neurons through peripheral inflammation and central inflammation. LPS seems to play an essential role in peripheral inflammation of ALS patients. The results of serum analysis showed that plasma LPS levels in sALS patients were significantly higher than healthy individuals, but none of the ALS patients participating in the study had any active infection during the trial [53]. Therefore, we speculate that the high plasma LPS level in patients with ALS is likely to come from the gut microbiota. This conjecture is also indirectly supported by the impaired intestinal barrier of SOD1^G93A^ mice [49]. An intact mucosal barrier is the first line of defense against pathogenic bacteria in the organism, and the increase of intestinal permeability is not conducive to the host's maintenance of internal balance. In addition, Qin and his colleagues found that after a single intraperitoneal injection of LPS, the microglial cells in wild-type mice will be chronic, voluntary, and progressive activated [93]. Nguyen et al. further found that a single acute intraperitoneal injection of LPS did not make a significant difference between SOD1^G93A^ and wild-type mice [94]. Only the chronic non-toxic dose of LPS injection resulted in accelerated motor axon loss and shortened median survival time in genetically susceptible mice, and none of these effects seen in wild-type mice. These experimental results provide us with a new idea that chronic LPS derived from gut microbiota is very likely an essential reason for the progression of ALS.

In a healthy body, the blood-brain barrier has very low permeability, and rarely can the peripheral LPS directly enter the brain. Therefore, LPS is more likely to have an indirect effect on neuroinflammation[95]. The research by Burberry et al. found that chemical analogs of microbial components could stimulate bone marrow-derived macrophages of C9orf72 mice [92], indicating that there might be other links between peripheral LPS and central system inflammation. Not only in animal models, Zhang R et al. also observed activated monocytes/macrophages in plasma of sALS patients [96], and further reported that the activation level of monocytes in sALS patients was directly related to plasma LPS levels four years later [53]. In addition, many other teams have repeatedly mentioned in their research results that there are proinflammatory cytokines released by macrophages such as TNF-α and IL-6 in the cerebrospinal fluid and serum of ALS patients [97, 98]. In summary, we speculated that products of gut microbiota represented by LPS could continuously and slowly enter the bodies of ALS patients, and the damage of intestinal barrier facilitated this process. LPS can activate monocytes and macrophages in peripheral blood to induce the release of a large quantity of pro-inflammatory cytokines, thereby affecting the CNS. In addition to increased proinflammatory cytokines, decreased secretion of anti-inflammatory cytokines such as IL-10 and inflammatory regulatory products such as butyric acid may also promote the progression of ALS [53, 99].

TNF-α may be a bridge between peripheral inflammation and central inflammatory response. The level of TNF-α in cerebrospinal fluid of ALS patients is increased[100], and animal experiments show that the increase of TNF-α in brain lags behind that of liver and serum [93]. Therefore, the production of TNF-α in the brain may be initiated after peripheral TNF-α enters the CNS through blood circulation. TNF-α entering the CNS may promote microglia-mediated neuronal death. Microglia is a type of glial cells, which is equivalent to macrophages in the brain and spinal cord. Microglial cell-centered neuroinflammation can cause neuronal death directly or indirectly and aggravate the symptoms of the disease. The role of TLR2-mediated signaling pathway in neuronal death has begun to receive more attention. It is found that chronic LPS stimulation can activate TNF-α gene and continuously induce the expression of TLR2 in microglia [94]. TLR2 can enhance the inflammatory response of microglia in the early stage while inducing microglia apoptosis in the late stage [101, 102]. In addition, motor neurons death mediated by FasR which belongs to TNF receptor superfamily, may also be one of the mechanisms of motor neuron death in ALS patients. Fas-induced neuronal death is realized through Fas-Daxx-ASK1-p38-NO-nNOS-peroxynitrite pathway.

Interestingly, the study also found that the SOD1^G93A^ mutant is highly sensitive to the activation of NO downstream pathway induced by Fas[103], which may explain the role of heredity in the development of ALS. As stated above, the neuronal damage mediated by TLR2 and FasR also supports the key bridging role of TNF-α. In addition, the dysfunction of DNA damage response (DDR) in microglia is also closely related to neurodegeneration. Normally, TDP-43 positive inclusions only appeared in the nucleus. However, TDP-43 positive inclusion was found in the cytoplasm of microglia and MDMi (microglia-like cells induced by PBMCs from peripheral blood) of ALS patients, and all inclusion were abnormally phosphorylated [104]. Furthermore, DNA damage accumulation and impaired phagocytic function have been found in MDMi cells of patients with ALS [104]. Therefore, we speculate that TDP-43 in ALS patients cannot be recruited in time to the foci of DNA damage in the nucleus. Dysfunctional DDR results in the accumulation of DNA mutations in neurons or microglia, ultimately causing abnormal neuroprotein synthesis and cell death.

NLRP3 inflammasome is an essential part of the innate immunity of the body after tissue damage [105]. Recent research suggests that NLRP3 inflammasome may also promote neuronal death in ALS patients. It has been found that NLRP3 and IL-1β could already be detected in SOD1^G93A^ mice in the pre-symptom stage, and this phenomenon was more obvious in 14-week-old animals [106]. Behavioral and cognitive abnormalities in ALS patients are thought to be associated with neurodegeneration in the subcortical region. NLRP3 expression was increased in both dorsal thalamic nucleus and neurons of SOD1^G93A^ mice [107]. It was also found that down-regulating the steroid hormone 17β-estradiol activated by inflammatory bodies can reduced motor neuron death in SOD1^G93A^ mice [108]. Increased levels of NLRP3, ASC, IL-18 and caspase-1 have been observed not only in animal models, but also in human patients with ALS [106, 109, 110].Therefore, NLRP3 inflammasome may become a potential target for intervention of neuronal death. The activation of NLRP3 inflammasome can be divided into two stages: initiation and activation [111]. Risk signals such as oxidative stress, lysosomal damage and Ca^2+^ mobilization can be identified through the damage-related molecular pattern (DAMPs) or the pathogen-related molecular pattern (PAMPs), thereby initiating the assembly of NLRP3 inflammasome [112]. Activated caspase-1 can generate cell membrane pores and initiate cell apoptosis [111]. Trimethylamine oxide (TMAO) and short-chain fatty acids (SCFAs) are important metabolites of intestinal microorganisms. By promoting the expression of TLR4, TMAO can activate the NLRP3 inflammasome, which increases the level of inflammatory factors in the CNS [113]. SCFAs, on the other hand, could inhibit the over-expressions of ASC, NLRP3, IL-18, IL-1β and caspase-1, showing the opposite effect to that of TMAO [114]. Activation of NLRP3 inflammasome can regulate caspase-1-dependent pathways and the release of pro-inflammatory cytokines such as IL-1β to respond to cell stress and infection. However, persistent neuroinflammation and brain injury will occur when NLRP3 inflammasome is over-activated [85], therefore aggravate the symptoms of the disease.

It has been widely recognized that the innate immune response is involved in the process of ALS, but whether adaptive immunity participates in the degeneration and death of motor neurons is still controversial. Burberry et al. reported neutrophils and T-lymphocyte infiltration in the spinal cord of C9orf72 mice [92], while Nguyen's team failed to detect CD8+ and CD4+ cells after immunohistochemistry of the spinal cord and brain of SOD1^G93A^ mice [94]. However, due to the differences between the two groups of experimental animal models, the interference of genetic factors in the experimental results cannot be excluded.

Through immune pathways, the CNS can be influenced by the gut microbiota, and the gut microbiota can also be affected by the CNS. The intestinal mucosal immune system is composed of intestinal mucosal epithelial tissues, mucosa-associated lymphoid tissues, immune cells and their secretions and symbiotic microbiota on the mucosa. It is an essential part of the immune system and the main place for local specific immune response. The Paneth cell, located at the base of the small intestinal crypt, is an intestinal epithelial cell that secretes defensins, which can penetrate the bacterial cell membrane to lyse bacteria and regulate the gut microbiota. Therefore, the dysfunction of Paneth cell will affect the steady state of gut microbiota. A study reported that the number of abnormal Paneth cells in SOD1^G93A^ mice increased while the production of defensin 5α decreased. This result implies a diminished role for Paneth cells in maintaining intestinal microbial homeostasis [49]. In addition, studies have shown that the ratio of *Sclerotinia* to *Bacteroides* of mice with the caspase-1 gene knockout is significantly reduced [115], which means that activated NLRP3 inflammasomes may aggravate the imbalance of gut microbiota by regulating its composition. Furthermore, a study evaluating gastrointestinal health and fecal microbiota of patients with ALS found that the concentrations of inflammatory markers such as sIgA, calmodulin and eosinophils in the feces of patients increased [116], which indicated that adaptive immune response activated in ALS patients may also pose challenges to the survival of some gut microbiota.

### 4.3 Endocrine pathway of brain- gut-microbiota axis

SCFAs, NAD+ precursors, amino acid derivatives and bile acids are all small molecules derived from gut microbiota [117]. In the following sections, we will focus on the recent findings of SCFAs and NAD+ precursors in ALS.

### 4.3.1 SCFAs

SCFAs mainly include propionate, acetate and butyrate. Most SCFAs come from fermentation of undigested fiber by colonic and cecal microbiota, with a small proportion are produced through amino acid metabolism [118]. After entering the bloodstream through uptake of intestinal epithelial cells, SCFAs can even enter the CNS through the blood-brain barrier [118].SCFAs can enter cells through passive diffusion, the MCT1 transporter, the SMCT1 transporter and the bicarbonate co-transport pathway [119]. After entering cells, SCFAs can act as histone deacetylase (HDAC) inhibitors or participate in cellular energy metabolism to regulate cellular functions. SCFAs can also induces a series of intracellular reactions by binding to cell surface receptors GPR41(FFAR3), GPR43(FFAR2) and GPR109A(NIACR1) [120, 121].

SCFAs can reduce intestinal barrier permeability by affecting the mucus layer and cell layer. SOD1^G93A^ mice showed increased intestinal barrier permeability and decreased intestinal butyric acid-producing bacteria. However, after butyrate treatment, scientists found that increased expression of the tight junction protein in the mice delayed the occurrence of ALS [49, 99]. Multiple studies have demonstrated that butyrate helps maintain the intestinal barrier's stability. Butyrate stimulates the expression of MUC-2 in goblet cells by triggering the release of prostaglandin E1/E2 from subepithelial myofibroblasts [122]. MUC-2, as an important glycoprotein in the mucus layer, isolates pathogenic bacteria and controls inflammation [123]. MUC-2 can also form the receptor complex of galectin-3-Dectin-1-FCGR2B and activate β-catenin to inhibit nuclear factor kB (NF-kB), thereby inhibiting the immune response of inflammatory dendritic cells (DC) [124]. For the cell layer, butyrate can promote the intestinal epithelial cells to consume oxygen, produce the hypoxia-inducible factor (HIF) and induce the expression of claudin-1 [125, 126]. In addition, butyrate can upregulate the actin-binding protein synaptophysin (SYNPO) in epithelial cells by inhibiting HDAC. Located at the tight junction of the intestinal epithelium and within F- actin fibers, SYNPO helps maintain the integrity of the intestinal barrier and cell movement. SYNPO-deficient mice have increased gut permeability and are vulnerable to colitis, and butyrate supplementation contributes to symptomatic improvement [127].

SCFAs regulate the body's inflammatory response through multiple pathways, the most important of which is through regulatory T cells (Tregs) and microglia. Tregs can inhibit pro-inflammatory T cells, activated macrophages and microglia, thus effectively controlling the peripheral and central inflammatory response [128]. It is proved that the quantity of Tregs in patients with faster progression of ALS is decreased, with a decrease in the expression of FOXP3 which is an important transcription factor for Tregs to function [128, 129]. A study by Furusawa et al. showed that butyrate derived from the microbiota could inhibit HDAC and up-regulate the expression of FoxP3, thereby inducing the differentiation of Tregs [130]. In addition, the binding of butyric acid to GPR 109 A (which is also a nicotinic acid receptor) can also induce the differentiation of Tregs [131]. Central inflammation caused by abnormal activation of microglia is an important pathogenesis of ALS [52]. Inhibition of HDAC in microglia by SCFAs reduced the release of pro-inflammatory cytokines (IL-6, IL-1β and TNF-α) and increased the release of anti-inflammatory cytokines (TGF-β and IL-4), thereby inhibiting inflammation [132, 133]. Ionized calcium-binding adaptor molecule 1 (IBA1) is a calcium-binding protein specifically expressed by macrophages and microglia. IBA1 is upregulated when microglia are activated. Butyrate supplementation has been shown to negatively regulate the serum IL-17 and LPS levels of SOD1^G93A^ mice and inhibit the expression of IBA1 in the spinal cord [134]. This study further sheds light on the mechanism by which butyrate improves ALS disease progression by inhibiting neuroinflammation.

SCFAs can act directly on the ENS as well as the ANS. GPR41/43 is an SCFAs receptor which is expressed in the myenteric plexus, the nodose ganglia and the dorsal root ganglia. When SCFAs act on the above receptors, the discharge of vague afferent nerves will be increased, the spinal cord will be excited, and eventually the intestinal activity will be enhanced [135, 136]. SCFAs can also act on the sympathetic nervous system. Muller and his colleagues found the regulatory effect of SCFAs on sympathetic ganglia and its neural circuit for promoting intestinal motility [137]. In addition, by increasing the proportion of cholinergic neurons in ENS, butyrate can also enhance the locomotor function of the colon [138]. The potential association between reduced SCFA-producing bacteria and disturbed intestinal motility in ALS patients may be associated with the above mechanisms. Unfortunately, the improvement effect of SCFAs on intestinal motility was mostly reflected in animal experiments, and no human trial has yet shown that SCFAs supplementation can significantly improve human intestinal motility [139, 140].

In conclusion, SCFAs can affect ALS in many ways. Drug design and research for SCFAs targets will have a broad prospect [141]. Recent clinical trials have shown that SCFA supplementation or high SCFA diet has a certain effect on improving ALS-related symptoms [142, 143]. However, the effect of SCFAs on improving the core symptoms of ALS needs further study.

### 4.3.2 NAD+ precursor

NAD+ precursors include nicotinamide nucleoside (NR), nicotinic acid (NA) and nicotinamide (NAM). NAD+ is an essential coenzyme for cellular energy transduction and antioxidant function. It is synthesized in the human body mainly through three pathways: de novo synthesis using dietary tryptophan (kynurenine pathway), de novo synthesis using dietary NA (Preiss-Handler pathway), and salvage synthesis using NAM [144]. Metabolic disorder and decreased level of NAD+ is closely related to ALS [144, 145]. ALS patients were found to have high levels of tryptophan and low levels of NAM in serum and cerebrospinal fluid, which indicated that there may be injuries in both the kynurenine pathway and salvage synthesis pathway, resulting in NAD+ deficiency and weakened antioxidant capacity [50, 144]. The main reason for the decrease of NAD+ content may be the deficiency of salvage synthesis [146]. Restoration of NAD+ level can ameliorate ALS from multiple perspectives, such as reducing the toxicity of astrocytes to neurons, promoting neurogenesis, enhancing muscle function and regulating gene expression [145]. In recent years, it has been found that the gut microbiota is conducive to maintaining the level of NAD+ [147]. Colonization of *A. muciniphila* of SOD1^G93A^ mice can increase NAM in the plasma and cerebrospinal fluid, resulting in improved ALS symptoms and prolonged survival [50]. The same experiment also showed that supplementing NAM can down-regulate the expression of ALS-related genes in the spinal cord of mice and improve the symptoms [50]. In a controlled trial, bacterial genes associated with NAM metabolism were reduced in stool and NAM levels in cerebrospinal fluid and serum were significantly reduced in ALS patients [89]. It was deduced from the above results that NAM derived from the microbiota could enter the CNS and affect the progression of ALS.

NAD+ precursor can mediate bidirectional communication between the host and gut microbiota. The intestinal microbiota can synthesize NA using NAM in the host's blood circulation, and NA can then return to the blood circulation for the host to synthesize NAD+. In germ-free mice, elevated levels of NAM in the intestinal lumen were accompanied by a decrease in NA levels. Furthermore, with intact gut microbiota, NA levels in blood circulation remained stable even when mice did not consume NA from the diet [148]. Therefore, the imbalance of gut microbiota may lead to the imbalance of NAD+ metabolism. In addition, gut microbiota may promote NR transformation. NR (vitamin B3) is a common NAD nutritional supplement with a stronger ability to produce NAD+ than NAM and NA [114].Compared to antibiotic-treated mice, mice with intact gut microbiota have a greater capacity to generate tissue NAD+ using NR [148]. Treatment of SOD1^G93A^ mice with NR can clear the mistakenly accumulated hSOD1 protein in mitochondria and induce neurogenesis [146]. Interestingly, in this experiment, NR treatment improved the locomotor ability of the mice but fail to delay the occurrence of ALS and extend the survival time, which is different from the results of the above-mentioned study supplemented with the NAM-producing strain *A. muciniphila*. Whether the microbiota imbalance in SOD1^G93A^ mice drives this outcome requires further investigation.

## 5. Potential treatment

**Figure 3.**
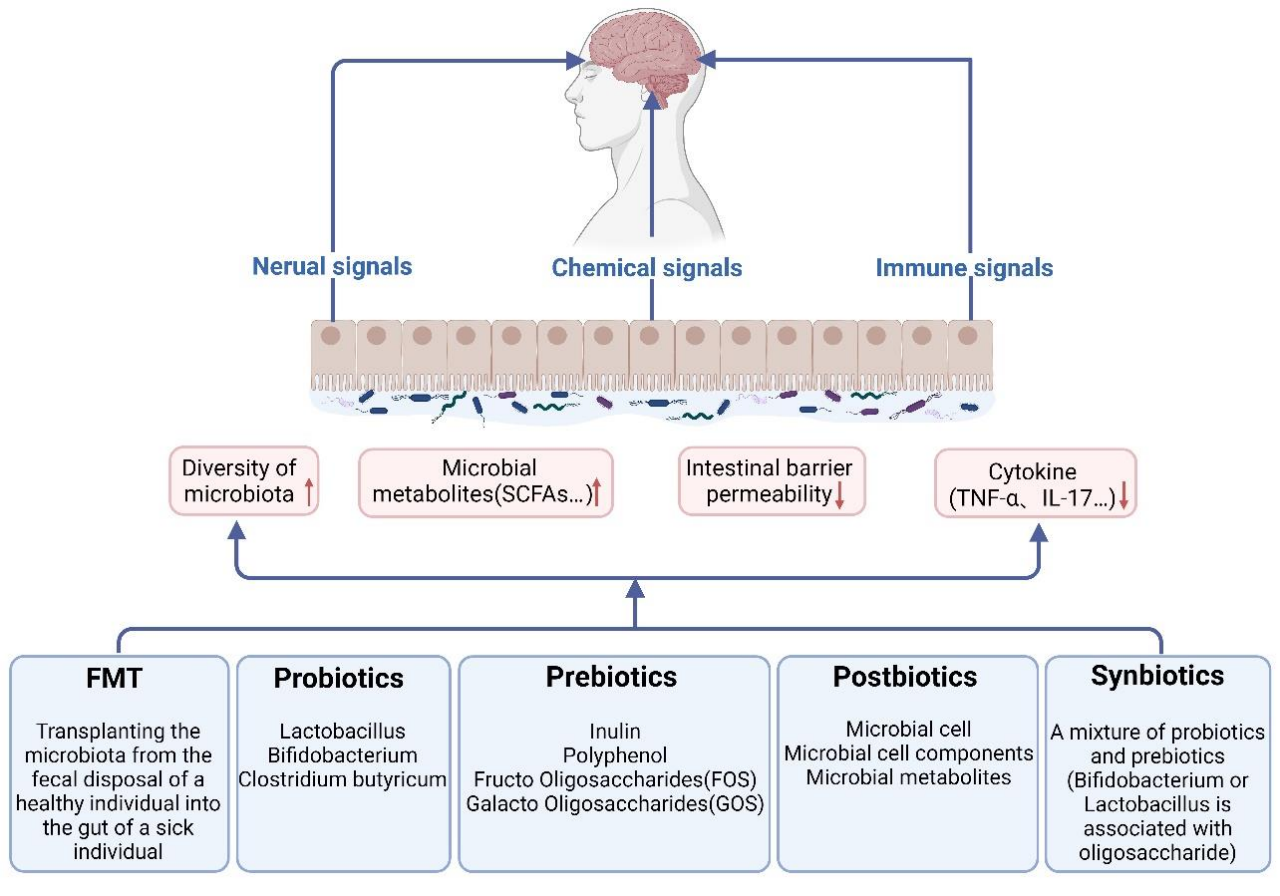
**Potential therapeutic strategies based on brain-gut-microbiota axis for ALS**. Potential therapeutic strategies for ALS include supplementation with probiotics, prebiotics, synbiotics, postbiotics and FMT. Based on the association of the brain-gut-microbiota axis with ALS, regulating the intestinal microbial composition or interfering with the microbiota metabolites can improve the pathology of ALS and delay disease progression by regulating the neural, immune and endocrine pathways. To date, most relevant studies have been conducted on animal models. The conversion between the research results on animal models and the treatment effect of human patients still needs to be further investigated.

Currently, only three drugs have been approved by the FDA for clinical treatment of ALS, namely, Riluzole, Edaravone, and Relyvrio [149, 150]. With increasing research on the relationship between gut microbiota and CNS diseases, many researchers hope to treat ALS by regulating the gut microbiota. Currently proposed potential therapeutic strategies mainly include FMT, probiotics, prebiotics, synbiotics and postbiotics (Fig. 3, Table 3). However, most of the research has been done in animal models. Few experimental data are available on patients. Whether the same conclusions can be switched between animal models and patients still needs to be further investigated.

### 5.1 FMT

FMT refers to the directional transplantation of microbiota from fecal of healthy individuals into the intestines of diseased individuals, reestablishing the intestinal microecology of patients and treating diseases [151]. Based on the function of gut microbiota in neurodegenerative diseases, FMT has shown promising therapeutic effects in the treatment of various central nervous system diseases such as Alzheimer's disease (AD) and Parkinson's disease (PD) [24]. FMT is a promising research direction for ALS treatment. However, there are not many clinical trials to verify the efficacy of FMT for ALS disease. A team conducted a multi-center randomized double-blind clinical trial of FMT for ALS in 2019 [152], but no trial result is available yet.

Interestingly, experimental results comparing the effects of fresh and frozen fecal transplantation indicated that the ability of FMT to restore intestinal microecology in mice decreased with the extension of freezing time [153]. The same experiment also found that freezing reduced the intestinal colonization ability of some microorganisms [153]. Therefore, the influence of fecal storage conditions on the therapeutic effect of FMT is also worthy of attention.

### 5.2 Probiotics

Probiotics are active microorganisms which can bring health to the host [154]. Probiotics improve the immune, nervous, and endocrine functions of the host through their metabolites. In addition, supplying the probiotics also helps to maintain the intestinal microecological balance of the host. Therefore, probiotics are a relatively safe and potential treatment for ALS and other CNS diseases. Common probiotics include *Lactobacillus*, *Bifidobacterium*, *Clostridium butyricum*.

It is demonstrated that NAM in cerebrospinal fluid of SOD1^G93A^ mice was significantly decreased. After oral administration of *Akkermansia muciniphila*, the content of NAM in cerebrospinal fluid is effectively increased and the movement ability of SOD1^G93A^ mice is effectively improved [89]. At the same time, another study also proved that colonization of *Akkermansia muciniphila* in SOD1^G93A^ mice can increase the NAM level in cerebrospinal fluid and improve the locomotor ability and survival rate of mice [50]. In addition, after patients with ALS were treated with a probiotic formula consisting of *Lactobacillus salivarius, Lactobacillus plantarum, Lactobacillus delbrueckii* subsp. *delbrueckii, Lactobacillus fermentum* and *Streptococcus thermophilus*, the diversity of gut microbiota increased [155].

### 5.3 Prebiotics

Prebiotics are non-digestible food ingredient which are beneficial to the host through selective modulation of microbiota [156]. It is often served as an alternative to probiotics or as an additional supplement. Administration of appropriate prebiotics can regulate the microecological balance of the host. At present, the common prebiotics include inulin, polyphenol, oligofructose and galactooligosaccharides.

Polyphenols are prebiotics widely studied. Many substances belong to polyphenol category, such as quercetin, catechins, naringin, hesperidin, genistein, proanthocyanidin, baicalin, gallic acid, caffeic acid, resveratrol, pterostilbene and curcumin. After intake of quercetin for six weeks, the abundance of *Akkermansia muciniphila* in rat model was obviously increased [157]. The same result has also been reported in a mouse after ingestion of green tea, and it was later demonstrated that the increase in *Akkermansia muciniphila* level was mainly affected by the polyphenol substance EGCG in green tea [158]. At the same time, EGCG can prolong the life of SOD1^G93A^ mice and alleviate the symptoms of ALS disease [159]. EGCG can also selectively promote the growth of *Lactobacillus* and *Bifidobacterium*, and significantly increase the level of SCFAs [160].

Galactooligosaccharide (GOS) is a common prebiotic. After the SOD1^G93A^ mice were ingested with GOS and prebiotic yogurt rich in GOS, the life span of the mice was significantly prolonged, the loss of motor neurons was reduced, and the expression of inflammatory factors TNF-α and iNOS was inhibited [161].

**Table 3:** Potential therapeutic strategies based on brain-gut-microbiota axis for ALS.

	Experimental subject		Conclusion	Ref.
	Animal model	Human volunteer		
FMT				
Mandrioli et al	_	42 patients diagnosed with ALS	No research results	[152]
Probiotics				
Blacher E et al Gotkine et al	SOD1(G93A) mice	_	Improved movement ability and survival rate after oral administration of Akkermansia muciniphila; Increased content of NAM	[50][89]
Di Gioia et al	_	28 males and 22 females diagnosed with ALS (mean age 60.24 years); 28 males and 22 females' matched controls (mean age 53.60 years)	After patients with ALS were treated with a probiotic formula consisting of five lactic acids, the diversity of the gut microbiota of the patients was regulated	[155]
Prebiotics				
Etxeberria et al	Wistar rats	_	Increased abundance of Akkermansia muciniphila in the intestinal tract after intake of quercetin for six weeks	[157]
Jeong et al	C57/BL6 mice	_	Increased abundance of Akkermansia muciniphila in the intestinal tract after a period of green tea intake	[158]
Song et al	SOD1(G93A) mice	_	Increased life span; decreased loss of motor neurons; inhibited expression of inflammatory factors TNF-α and iNOS after intake of GOS and prebiotic yogurt rich in GOS	[161]
Postbiotics				
Zhang et al	SOD1(G93A) mice	_	Significant recovery of the intestinal barrier function; delay in the progress of ALS disease; increased life span after butyrate treatment	[99]
Ogbu et al	SOD1(G93A) mice	_	Obviously decreased of inflammatory factors such as IL-17 and LPS after butyrate treatment	[134]
Kaji R et al	_	260 patients diagnosed with ALS	Prolonged survival and improved the symptoms of ALS patients (diagnosed within 1 year of symptom onset) after treatment with ultra-high doses of mecobalamin	[166]
Oki R et al	_	126 patients diagnosed with ALS (completed the observation period and double-blind stage)	Prolonged survival and improved the symptoms of ALS patients (diagnosed within 1 year of symptom onset) after treatment with ultra-high doses of mecobalamin	[167]

### 5.4 Synbiotics

Synbiotic is the mixture of probiotics and prebiotics. Synbiotics supplementation is conducive to the interaction of probiotics and prebiotics, which can better regulate the microecology of gut microbiota and bring benefits to the host [162]. To some extent, synbiotics supplementation should provide better results than supplementation with either probiotics or prebiotics alone. The most common synbiotics are mainly composed of *Bifidobacterium* or *Lactobacillus* paired with oligofructose. There have been few studies investigating the effect of synbiotics on ALS patients or animal models. However, there have been many studies related to synbiotics in other neurodegenerative diseases. Overall, synbiotics have beneficial effects on neurodegenerative diseases. Synbiotic treatment of AD mice model improves the cognitive symptom of mice and significantly reduces the level of inflammatory factor TNF-α [163].

### 5.5 Postbiotics

Postbiotics are bioactive compounds produced by food-grade microorganisms, including inanimate microbial cells, microbial cell components and metabolites [164, 165]. The common metabolites in postbiotics include vitamins, SCFAs, lipids and proteins. The common microbial cell components include peptidoglycan and teichoic-acid. After butyrate treatment of SOD1^G93A^ mice, the integrity of intestinal barrier was significantly improved, the progress of ALS was delayed, and their life span was prolonged [99]. Similarly, after butyrate treatment of SOD1^G93A^ mice, the inflammatory factors such as IL-17 and LPS in the mice serum were obviously reduced [134].

Vitamin B12, as a kind of postbiotic, can be converted into methylcobalamin in the body. A Japanese clinical study of ultra-high dose mecobalamin in ALS patients found that mecobalamin at an ultra-high dose did not show significant efficacy in the entire study population [166]. However, this experiment also showed that in patients diagnosed within one year of symptom onset, treatment with ultra-high doses of mecobalamin has shown to prolong survival and improve the symptoms of ALS [166]. Moreover, Oki and his colleagues further validated the efficacy of ultra-high dose mecobalamin in patients with early ALS and reached the same conclusion [167].

## 6. Future directions

As for the regularity of gut microbiota changes in ALS patients, no unified conclusion has been reached by various teams. We hope that more scientists will participate in the research of this issue. Such an investigation may lead to successful localization of one or several microbes that are specifically altered in ALS patients. Thus, combined analysis of the changes of ALS-related microbiota through fecal microbial detection may be able to predict the risk of ALS, diagnose or evaluate ALS patients. In addition, after clarifying the effects of various microbial changes on ALS, we can supplement certain beneficial bacteria through fecal transplantation and other means, restore the gut microbiota in ALS patients and alleviate the progression of ALS. In addition, we found that the differences in gut microbiota of ALS patients of different ages are currently in the preliminary stage of research. More efforts are needed to explore the characteristics of gut microbiota in ALS patients at different ages.

The effect of antibiotics on different genotypes in animal models is also of great interest. SOD1^G93A^ and C9orf72 mice showed distinct responses to broad-spectrum antibiotics [50, 52]. However, because the broad-spectrum antibiotics and animal models used by the two teams in animal experiments are different, it is not clear what factors lead to the opposite effects. This would be a very interesting study, because the results of this experiment could in part reveal the function of genetic factors in regulating the gut microbiota. If genetic factors can indeed affect the composition of gut microbiota in ALS patients to a large extent, we may need to pay more attention to the pathogenic genes of ALS patients in the future and use specific drugs according to the patient's genotype.

Current studies have found that the intestinal, blood-spinal cord and blood-brain barrier function are damaged in ALS patients and animal models. Furthermore, the results of animal experiments show that barrier function damage happens before the occurrence of ALS [60]. Broken barrier function may play a key role in ALS. Therefore, it is significant to clarify whether the impairment of barrier function is the cause or the pathogenesis of ALS, and what factors lead to the impairment of barrier function.

Unlike neurons in the CNS, neurogenesis occurs in adult enteric neurons [88]. To what extent can the altered gut microbiota affect the function and structure of enteric neurons in ALS patients? Moreover, to what extent do enteric neuronal abnormalities affect intestinal motor function in ALS patients? These are questions that both deserve further exploration. In addition, whether adaptive immunity is involved in neuronal degeneration and death is also a worthy research direction. For the answer to this question, the experimental results of different experimental teams give different answers, and there is no pathological study report related to human ALS patients. Our review summarizes potential therapeutic strategies for ALS. Most of these therapies have only been tested in animal models of ALS and have not yet entered the clinical trial stage. In the future, we look forward to more scientists participating in to further clarify the therapeutic effect of gut microbiota intervention on ALS animal models and promote the development of ALS treatment strategies.

Finally, we can find that most of the current research results on ALS are experimental conclusions obtained on animal models. The key to benefit ALS patients is to further explore the advantages and disadvantages of various animal models of ALS and to strengthen the translation between experimental results of animal models and human diseases.

### Conclusion

Brain-gut-microbiota axis is a sophisticated interaction system, including neural, immune, and endocrine networks. Accumulating evidence suggests that the gut microbiota plays an essential role in ALS. Therefore, the brain-gut-microbiota axis provides a new direction to elucidate the pathogenesis of ALS. This paper summarizes the dysbiosis of gut microbiota found in ALS animal model and patients recently and tries to clarify the relationship between gut microbiota and pathological changes of ALS. However, the exact mechanism of the role of the brain-gut-microbiota axis in ALS is still unclear. Further studies are needed to clarify the role of gut microbiota in the progression of ALS in the future. In addition, strengthening the transformation between the results of animal models and human diseases is also a very critical work.

Further understanding of the relevance between gut microbiota and ALS may help the diagnosis and therapy of ALS, ultimately benefiting patients. Although therapeutic methods such as FMT, prebiotic supplements and probiotics are still at the primary stage of research, these therapeutic strategies are likely to bring new hope to ALS patients based on the therapeutic mechanism of restoring intestinal microbial homeostasis.
